# Supplementary material for: Envelope glycoprotein mobility on HIV-1 particles depends on the virus maturation state
Source: Nat Commun. 2017 Sep 15;8:545. doi: 10.1038/s41467-017-00515-6 (PMC5601426; doi:10.1038/s41467-017-00515-6)
Supplement: Supplementary file 1 — Supplementary Information [file 41467_2017_515_MOESM1_ESM.pdf]

File name: Supplementary Information
Description: Supplementary Figures and Supplementary Methods

File name: Peer Review File

Supplementary Information

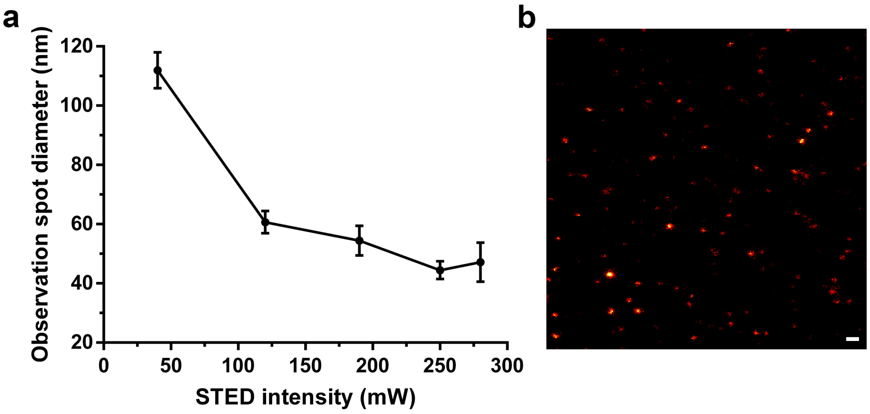

Supplementary Figure 1 | STED microscope calibration. (**a**) Mean ± SD of the observation spot diameter at different STED laser powers was determined by STED-FCS calibration measurements of DPPEKK114 diffusion in supported lipid bilayers. Results represent 30 measurements each from 3 separate preparations. For the virus measurements a minimum STED laser power (≈180 mW) was set to generate an observation spot of less than half in diameter than the size of the HIV-1 particles (140 nm diameter) and an acceptable signal-to-noise ratio. (**b**) STED microscopy imaging of 20 nm fluorescent Crimson beads gives an estimate of the microscope's spatial resolution. Mean bead diameter FWHM = 45 nm at 250 mW STED intensity. Scale bar: 200 nm.

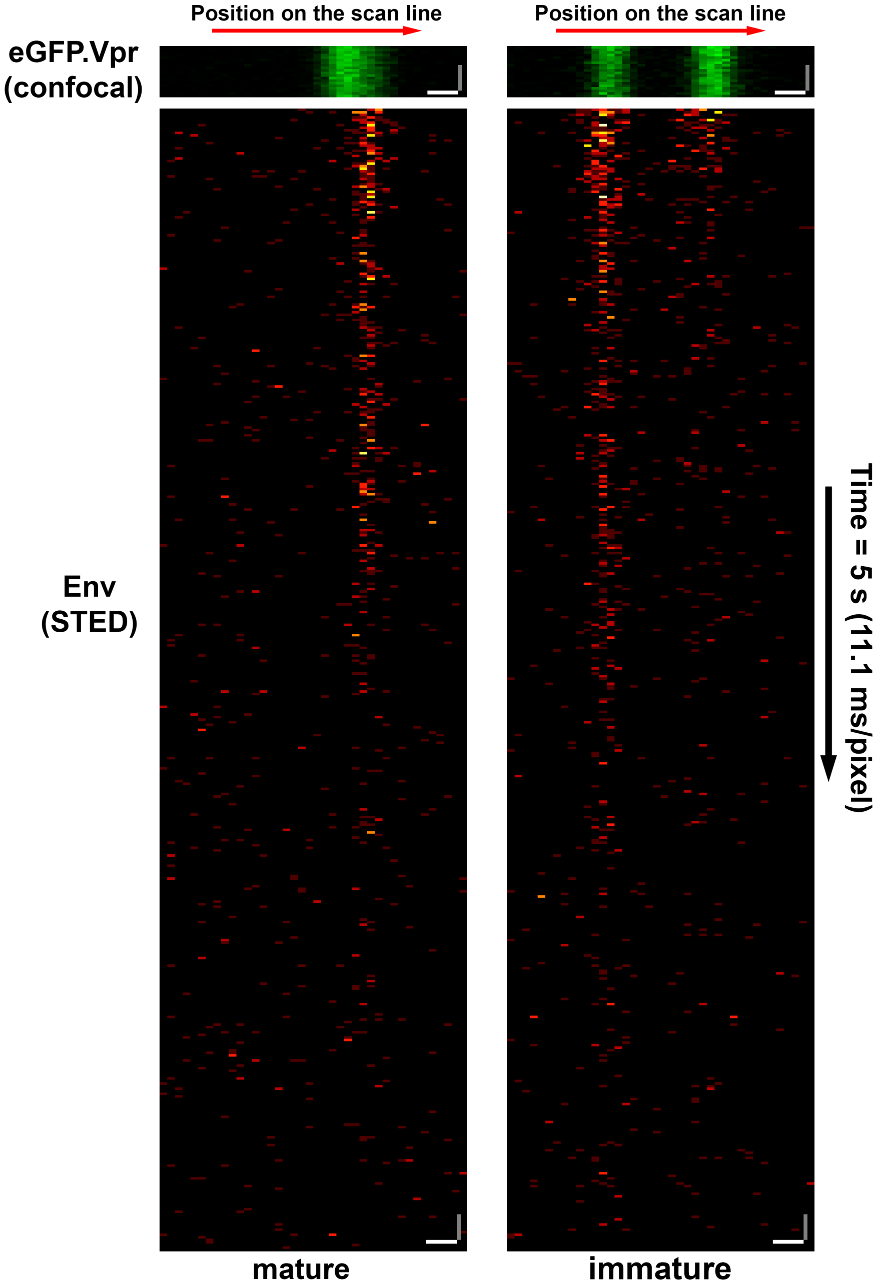

Supplementary Figure 2 | sSTED-FCS intensity carpets of mature and immature HIV-1 particles. Representative signal intensity carpets for eGFP.Vpr (green, confocal) and Env (orange, STED) on mature and immature HIV-1 particles. Image x- and y-axis correspond to the position on the scan line and signal intensity signal intensity at each time point, respectively. Y-axis was down-sampled from 0.9 kHz to 90 Hz to fit images on the page. Scale bars: x-axis (white) = 200 nm, y-axis (grey) = 111 ms.

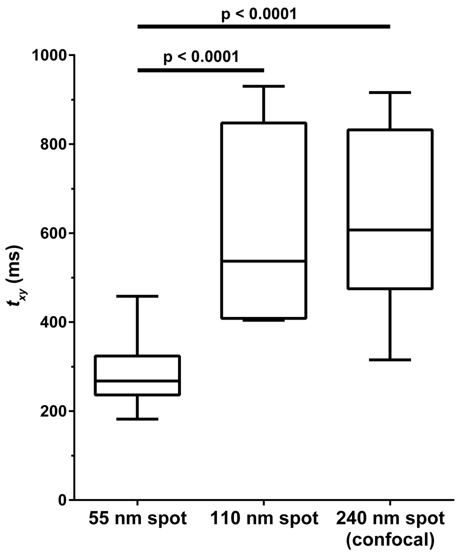

Supplementary Figure 3 | HIV-1 surface Env mobility can only be detected with 55 nm observation spot. Median Env transit times (t_{xy}) through the different diameter observation spots were determined by sSTED-FCS measurements of 50 mature virus particles each from two independent virus preparations. Results are shown in a box (IQR) and whisker (min/max) plots. Very high transition times (t_{xy} > 400 ms) for 110 nm and 240 nm observation spots are similar to values of t_{xy} determined for PFA fixed particles, which were ruled by photobleaching only (compare Supplementary Fig. 4). Consequently, observation spots with diameters as small as 55 nm in diameter are required for the determination of Env mobility on virus surface. sSTED-FCS experiments using even smaller observation spots were not feasible due to too low signal-to-noise ratios.

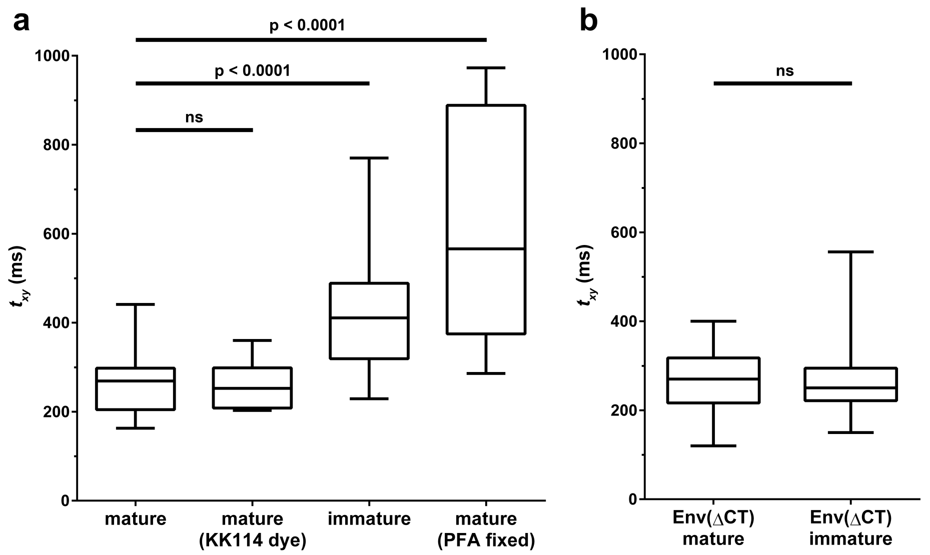

Supplementary Figure 4 | HIV-1 Env transit times. Median Env transit times (t_{xy}) through the 55 nm observation spot were determined by sSTED-FCS measurements of 50 particles each from two independent virus preparations. Results are shown in a box (IQR) and whisker (min/max) plot and the statistical significance was assessed by Wilcoxon rank-sum test. (**a**) t_{xy} values of mature, immature and mature particles labelled with an alternative dye (KK114) as well for PFA fixed mature particles. (**b**) t_{xy} values of mature and immature particles pseudotyped with Env(ΔCT).

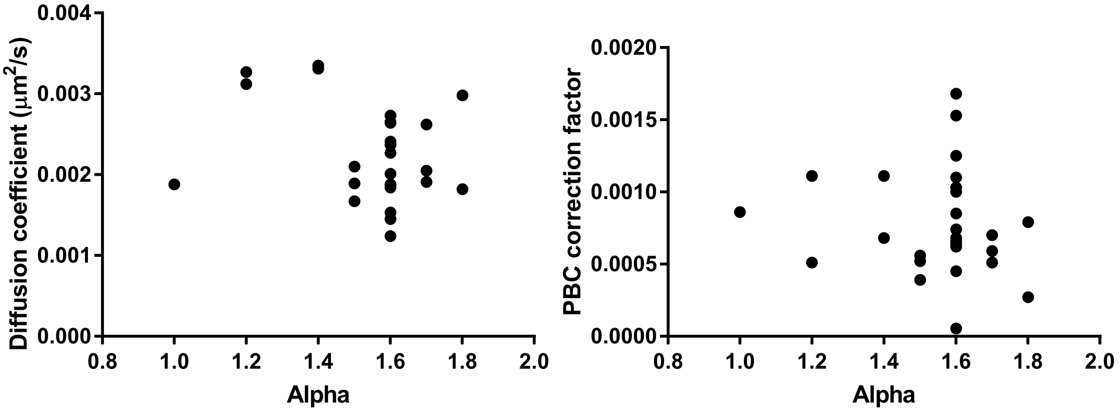

Supplementary Figure 5 | The degree of apparent anomalous diffusion is independent from calculated diffusion coefficients and the degree of photobleaching. Median values of the anomaly parameter α (following correlation curve fitting using 2D diffusion model) as a function of corresponding diffusion coefficients or photobleaching (PBC) correction factors for individual Env diffusion correlation curves on mature HIV-1 particles. No correlation is visible.

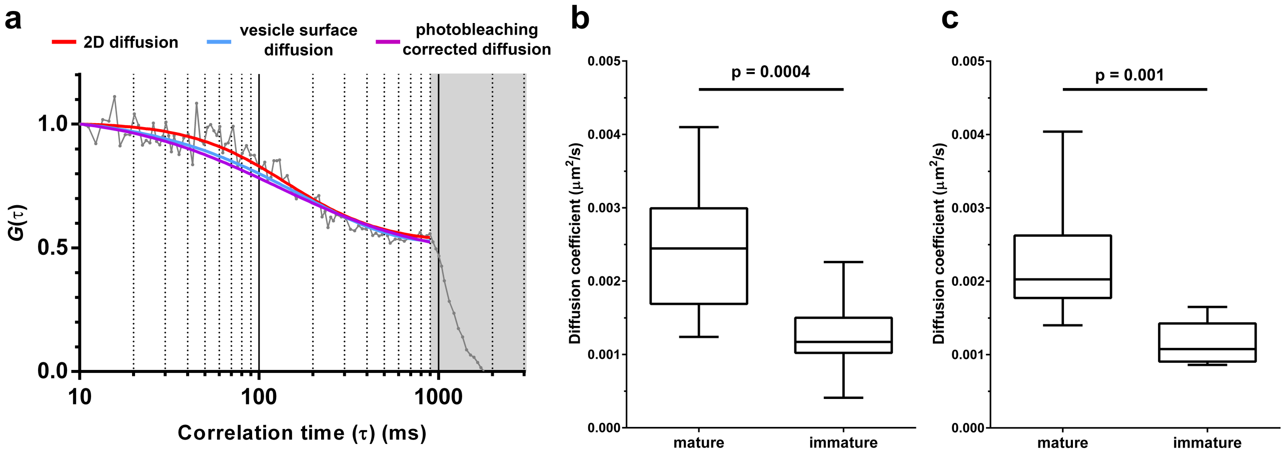

Supplementary Figure 6 | Alternative correlation curve fitting models for the determination of Env molecular mobility on HIV-1 surface. (**a**) Representative correlation curves of Env diffusion on a mature HIV-1 Env particle (black line) fitted with the generic 2D diffusion model (red), the vesicle surface diffusion model (blue) and the photobleaching corrected diffusion model (purple). The following parameters were fixed during the fitting: vesicle/virus radius = 70 nm and FWHM = 55 nm (determined by STED-FCS calibration) in the vesicle surface diffusion model, and $K_z = 0.025\ s^{-1}$ (determined from sSTED-FCS data on fixed HIV-1 particles) in the photobleaching corrected diffusion model. (**b** and **c**) Median Env diffusion coefficient (*D*) of mature and immature HIV-1 particles obtained from the vesicle surface diffusion model (**b**) and the photobleaching corrected diffusion model (**c**). Results are shown in a box (IQR) and whisker (min/max) plot and the statistical significance was assessed using Wilcoxon rank-sum test.

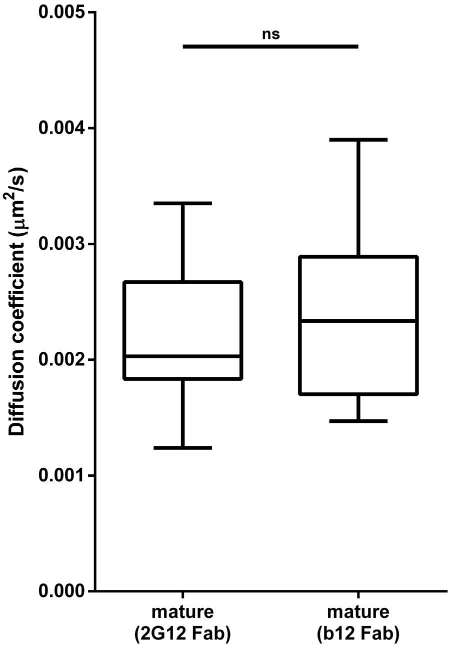

Supplementary Figure 7 | Comparison of diffusion coefficients of HIV-1 Env tagged with 2G12 and b12 anti-gp120 Fab fragment. Median Env diffusion coefficients (*D*) were determined by sSTED-FCS measurements of 50 mature particles each from two independent virus preparations. Env was stained with Abberior STAR RED (KK114)-labelled anti-gp120 2G12 or b12 Fab immunocomplexes. Results are shown in a box (IQR) and whisker (min/max) plot and the statistical significance was assessed by Wilcoxon rank-sum test.

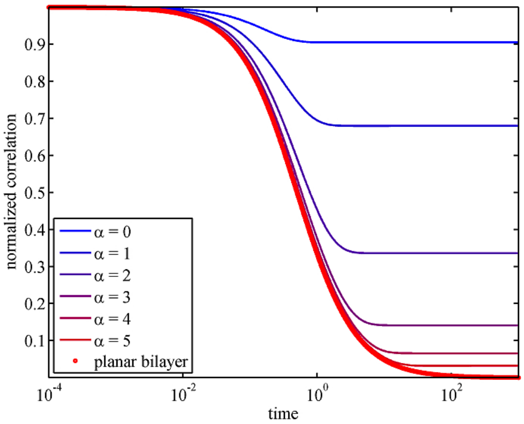

Supplementary Figure 8 | Autocorrelation functions for molecules diffusing in the membrane of a vesicle. See Supplementary Methods section below.

Supplementary Methods

Diffusion on the surface of a vesicle

We consider a fluorescence correlation spectroscopy (FCS) measurement on a fixed spherical vesicle. The autocorrelation function (ACF) is then given by

$$g(t) = \int_0^{\pi} \theta \sin\theta \int_0^{2\pi} d\phi \int_0^{\pi} \theta_0 \sin\theta_1 \int_0^{2\pi} d\phi_0 \quad U(\theta,\phi)\, G(\theta,\phi,t|\theta_0,\phi_0)\, U(\theta_0,\phi_0) \tag{1}$$

where $U(\theta,\phi)$ is the chance to excite and detect a photon from a molecule at angular coordinates θ and ϕ on the vesicle's surface (molecule detection function or MDF), and $G(\theta,\phi,t|\theta_0,\phi_0)$ is the chance that a molecule has moved to position θ and ϕ within time t if it was at position θ_0 and ϕ_0 at time zero (Green's function). For pure diffusion, G has to obey the two-dimensional diffusion equation

$$\frac{\partial G}{\partial t} = \frac{D}{a^2}\left(\frac{1}{\sin\theta}\frac{\partial}{\partial\theta}\sin\theta\frac{\partial G}{\partial\theta} + \frac{\partial^2 G}{\partial\phi^2}\right) \tag{2}$$

where a is the vesicles radius, and G has to obey the initial condition

$$G(\theta,\phi,0|\theta_0,\phi_0) = \frac{1}{\sin\theta}\delta(\theta-\theta_0)\delta(\phi-\phi_0). \tag{3}$$

It can be shown that Green's function has the explicit form

$$G(\theta,\phi,t|\theta_0,\phi_0) = \sum_{\ell=0}^{\infty}\sum_{m=-\ell}^{\ell} P_\ell^m(\cos\theta)P_\ell^m(\cos\theta_0)\frac{2\ell+1}{4\pi}\frac{(\ell-m)!}{(\ell+m)!} \cdot \exp\left[im(\phi-\phi_0) - (D/a^2)\ell(\ell+1)t\right] \tag{4}$$

where P_ℓ^m are the associated Legendre polynomials. Let us now specify the MDF $U(\theta,\phi)$. We assume that the focus is centered on the vesicle, and that neither excitation intensity distribution nor the point spread function of detection change much along the optical axis over the extension of the vesicle. Then, $U(\theta,\phi)$ can be approximated by a two-dimensional Gaussian,

$$U(\theta,\phi) = U(\theta) \propto \exp\left(-\frac{a^2\sin^2\theta}{2\sigma^2}\right) \tag{5}$$

where σ^2 is the variance of the Gaussian. In that case, the expression for the ACF simplifies to

$$g(t) = \sum_{\ell=0}^{\infty}(2\ell+1) \left[\int_{-1}^{1} P_\ell(x)\exp\left(\frac{a^2x^2}{2\sigma^2}\right)\right]^2 e^{-(D/a^2)\ell(\ell+1)t} \tag{6}$$

with P_ℓ being simple (non-associated) Legendre polynomials. Although the integrals within the square brackets can be solved analytically, they lead to increasingly complicated polynomials of the x-pre-factor in the exponential, and in practice, it is simpler to compute then numerically.

Typical examples are shown in the Supplementary Figure 8 for the numerical values $\sigma = 1/\sqrt{2}$, $D = 1$, and various values of $a = \sqrt{2^\alpha}$ with $\alpha = 0\ldots5$. Shown is also the $\alpha \leftarrow \infty$ limit, which is identical to the correlation function for a planar bilayer, $g(t) = 1/\left(1 + Dt/\sigma^2\right)$.
